# Retraction Note: Anatomical adjustment of mature leaves of sycamore maple (*Acer pseudoplatanus* L.) to increased irradiance

**DOI:** 10.1007/s11120-022-00940-9

**Published:** 2022-07-06

**Authors:** Tomasz P. Wyka, Piotr Robakowski, Roma Żytkowiak, Jacek Oleksyn

**Affiliations:** 1 Adam Mickiewicz University, Faculty of Biology, General Botany Laboratory, ul. Uniwersytetu Poznańskiego 6, 61-614 Poznań, Poland; 2 Poznań University of Life Sciences, Faculty of Forestry, ul. Wojska Polskiego 71a, 60-625 Poznań, Poland; 3 Polish Academy of Sciences, Institute of Dendrology, ul. Parkowa 5, 62-035 Kórnik, Poland

## Retraction Note to: Photosynthesis Research (2022) 152:55-71 10.1007/s11120-022-00898-8

The authors have retracted this article. Following publication, errors were identified affecting Figs. 4 and 6. The values of mean total chlorophyll concentration in control plants and individual leaves (Figs. 4a and 6a, respectively) reported in the article were several fold higher than those from other reports. Moreover, the chlorophyll a/b ratios of control plants presented in Fig. 4c were also much higher than reported values for tree seedlings. The authors believe these discrepancies are due to technical errors and intend to submit a revised manuscript which will undergo peer review. All authors agree with this retraction.

